# Patterns of Coke Adaptability and Evaluation Mechanisms Under Different Blast Furnace Atmospheres

**DOI:** 10.3390/ma19153340

**Published:** 2026-08-06

**Authors:** Qiang Li, Xuefeng She, Guang Wang, Qingguo Xue, Haibin Zuo, Jingsong Wang

**Affiliations:** State Key Laboratory of Advanced Metallurgy, University of Science and Technology Beijing, Beijing 100083, China

**Keywords:** hydrogen-rich blast furnace, coke, reaction start temperature, solution-loss behavior, softening-melting behavior

## Abstract

To optimize the configuration of low-carbon blast furnace raw materials and fuels, this study systematically evaluated the reaction initiation temperature, solution loss degradation, ferrous burden softening–melting behavior, and gas permeability of different reactive cokes under four simulated atmospheres: a traditional blast furnace (TBF), a hydrogen-rich traditional blast furnace (TBF-H_2_), an oxygen blast furnace (OBF), and a hydrogen-rich oxygen blast furnace (OBF-H_2_). The results show that with increasing coke reactivity, enhanced early reduction leads to a decrease in the softening initiation temperature, dropping from 1089 to 1074 °C under the traditional blast furnace atmosphere. In hydrogen-rich atmospheres, high concentrations of H_2_O significantly reduce the proportion of optically anisotropic structure—which characterizes coke strength—from 61.0% to 41.7%, indicating intensified degradation and fragmentation of the load-bearing coke structure. Process adaptability evaluations reveal that under the TBF atmosphere, burden-column permeability is highly sensitive to coke reactivity, with Coke 3 causing the maximum pressure drop (ΔPmax) to increase to 35.7 kPa, indicating the need for high-quality coke. Conversely, under OBF and OBF-H_2_ processes, the high reduction potential compresses the melting zone to a narrow range of 10–30 °C, maintaining both ΔPmax (11.5–14.0 kPa) and the cohesive-zone permeability index S (<150 kPa·°C) at consistently low levels. Within the present laboratory apparatus and the three-coke dataset, the oxygen-blast-furnace atmospheres reduced the sensitivity of cohesive-zone permeability to coke reactivity. This result suggests a potentially broader coke-quality window, but pilot-scale validation, process simulation, and economic assessment are required before industrial application or cost advantages can be established.

## 1. Introduction

In recent years, carbon dioxide (CO_2_) emissions have become a major global challenge. Because it depends heavily on fossil fuels such as coal and coke, the iron and steel industry is a major contributor to global CO_2_ emissions [1,2,3,4]. Reducing CO_2_ emissions during the ironmaking process is of paramount importance for the low-carbon transformation of the steel sector [5,6,7]. Consequently, novel blast-furnace technologies—such as the traditional blast furnace (TBF), hydrogen-rich TBF (TBF-H_2_), oxygen blast furnace (OBF), and hydrogen-rich OBF (OBF-H_2_)—have progressively become important development routes for energy conservation and emission reduction in the iron and steel industry [8,9,10]. In these emerging low-carbon technologies, the gas atmosphere inside the blast furnace undergoes a fundamental shift. The substantial increase in reduction potential, coupled with the higher water-vapor (H_2_O) concentration, poses new challenges to the high-temperature load-bearing capacity of coke, which serves as the skeleton of the burden column [11,12]. A detailed investigation of coke adaptability under various novel blast furnace atmospheres is therefore important for optimizing the configuration of low-carbon blast furnace raw materials and fuels and for ensuring smooth and stable blast furnace operation.

Existing studies remain largely confined to a single gas atmosphere or to gasification-induced degradation. A systematic comparison and a process-adaptability evaluation mechanism linking the intrinsic matrix properties and microstructural evolution of coke to macroscopic softening–melting–dripping behavior and gas permeability across different process atmospheres are still lacking [8,13,14,15,16,17,18,19,20,21,22,23,24,25,26,27,28]. Previous studies have demonstrated that, at the same temperature, the gasification rate of coke with H_2_O is significantly higher than that with CO_2_; H_2_O gasification also has a lower apparent activation energy and reaction initiation temperature [13]. This implies that, in a hydrogen-enriched blast furnace, coke gasification gradually changes from a system dominated by the Boudouard reaction (C + CO_2_ → 2CO) to one involving both the water–gas reaction (C + H_2_O → CO + H_2_) and the water–gas shift reaction (CO + H_2_O ⇌ CO_2 +_ H_2_). In particular, in the high-temperature zones of the middle and lower blast furnace, intensification of the water–gas reaction may initiate significant coke mass loss earlier and thereby alter the distribution of gasification within the burden column [14,15]. Lyu et al. [21] demonstrated that increasing coke reactivity lowers both the softening-start and melting-start temperatures while increasing the maximum pressure drop and maximum characteristic value. Sunahara et al. [22] reported that increasing coke reactivity narrows the high-temperature melting zone and lowers the dripping temperature, but increases gas-flow resistance in the blast furnace. Cheng et al. [23] found that higher coke strength after reaction (CSR) improves coke quality, enhances burden-column permeability, and reduces ΔPmax. Das et al. [24] similarly reported that higher CSR improves permeability and productivity while reducing the pressure drop and shortening the dripping stage. Kumar et al. [25] noted that higher CSR preserves coke strength in the cohesive zone, thereby improving gas and liquid permeability through the deadman and increasing overall smelting capacity. Meng et al. [26] performed cross-experiments using pellets with different reducibilities and cokes with different reactivities. Their results showed that a burden containing highly reducible pellets and highly reactive coke can lower the thermal-reserve-zone temperature, enhance gas utilization, and improve in-furnace permeability. Lan et al. [27] conducted cross-experiments under reducing atmospheres containing 0% and 10% hydrogen. They found that increasing coke reactivity promotes carburization of metallic iron but deteriorates blast furnace permeability. However, while the aforementioned studies individually explored specific phenomena such as coke reactivity, hydrogen enrichment, and softening–melting behavior, a significant scientific gap remains. There is still a lack of a systematic evaluation mechanism that correlates the evolution of coke micro-matrix and optical texture with macro-level burden softening–melting–dripping behavior and gas permeability under different blast furnace processes.

In this study, three cokes with representative reactivities were selected to investigate their behavior under four typical smelting process atmospheres: TBF, TBF-H_2_, OBF, and OBF-H_2_. First, the reaction initiation temperatures of the solution-loss reaction for different reactive cokes were measured using thermogravimetric analysis (TGA). Second, by employing a high-temperature gasification reaction apparatus and a compressive strength tester, combined with microstructure observation and quantitative characterization of the optical structure index (OTI), the degradation mechanisms of the coke skeleton microstructure induced by the novel blast furnace atmospheres were examined. Finally, the macro-level regulatory effects of coke reactivity on the softening–melting behavior and gas permeability of the iron-bearing burden were explored using a high-temperature softening–melting–dripping simulation apparatus. This work aims to clarify the process-specific adaptability of coke under various blast furnace atmospheres, thereby providing critical theoretical support for the optimized configuration and efficient utilization of raw materials and fuels in future low-carbon smelting.

## 2. Materials and Methods

### 2.1. Materials

The experimental raw materials used in this study were sourced from a domestic steel company. The pelletized ore used in the experiments was screened to a particle size range of 10.0–12.5 mm and dried for 2 h at 120 °C. The chemical composition is shown in Table 1. The industrial analysis of the cokes was determined in accordance with the standard GB/T 2001-2013 [29]. The CRI and CSR were evaluated using a standard high-temperature reactivity apparatus according to GB/T 4000-2017 [30]. The proximate analysis results, along with the CRI and CSR values, are detailed in Table 2.

The three selected cokes exhibit distinct property gradients and are representative of industrial coke. Coke 1 (CRI 20.7%, CSR 66.6%) represents high-strength premium coke used in large conventional blast furnaces; Coke 2 (CRI 26.4%, CSR 62.4%) represents typical commercial-grade coke; and Coke 3 (CRI 40.3%, CSR 45.2%) represents highly reactive, low-strength coke that is traditionally unsuitable for blast furnace operation. This broad range spans the quality boundaries of current and future low-carbon ironmaking burden designs, providing a clear benchmark to assess process tolerance to coke thermal strength.

### 2.2. Methods

#### 2.2.1. Reaction Start Temperature of Coke Solution Loss

First, the coke sample to be tested was crushed, and granular specimens with a particle size range of 74–100 μm were selected and dried in a drying oven at 105 °C for 2 h. During the experiment, a weighed amount of the dried coke sample was evenly spread across the bottom of a corundum crucible to ensure uniform heating of the sample and sufficient contact with the reactive atmosphere. Subsequently, the crucible containing the sample was placed onto the balance support of the thermogravimetric analyzer. High-purity argon gas (with a flow rate of 100 mL min^−1^) was initially introduced into the furnace chamber to purge any residual air inside the furnace tube, thereby eliminating its interference with the experiment. The furnace was then heated to 800 °C at a heating rate of 10 °C min^−1^ and held isothermally for 5 min to remove trace amounts of residual moisture and volatiles from the sample. Once the thermogravimetric baseline stabilized, the gas atmosphere inside the furnace was switched to the preset reactive atmosphere, with the total gas flow rate controlled at 100 mL min^−1^. The temperature was further raised to 1200 °C at the same heating rate of 10 °C min^−1^, while the sample mass and the corresponding temperature variations were continuously recorded throughout the process. The specific gas compositions are listed in Table 3. In this study, the start temperature of the dissolution reaction is defined as the temperature at which a 5% weight loss occurs.

#### 2.2.2. Solution Loss Reaction Behavior of Coke

The coke specimens utilized in the experiments were machined into pseudo-spherical particles with a diameter range of approximately 23–25 mm. The prepared specimens were dried at 200 °C for 2 h, cooled to room temperature, and subsequently screened using 23 and 25 mm sieves to remove any coke breeze adhering to the coke pieces.

**Figure 1:**
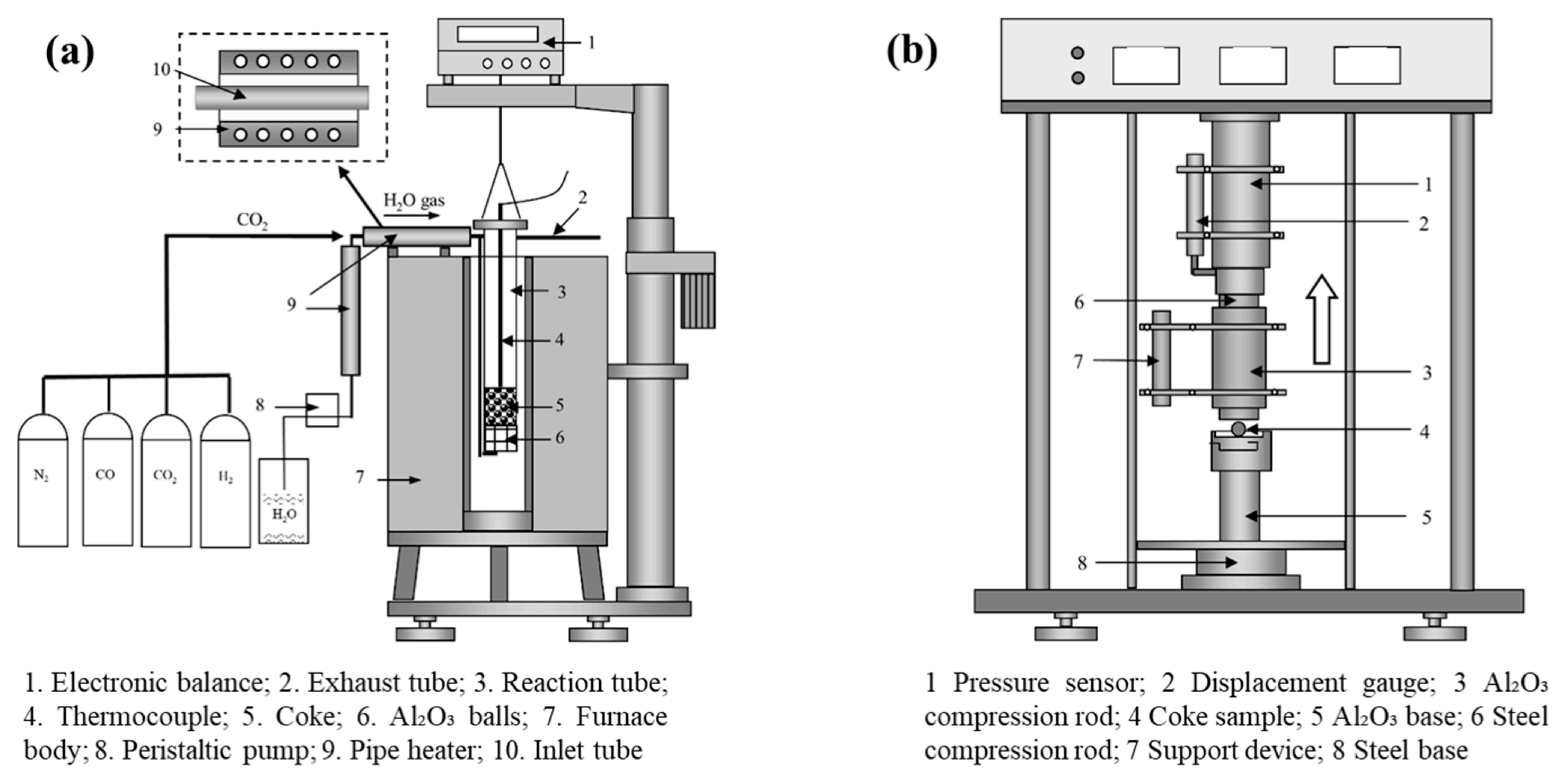
Experimental apparatuses: (**a**) Coke gasification reaction experimental setup. (**b**) Pressure measurement experimental setup.

The coke gasification reaction apparatus employed in this study is illustrated in Figure 1a. The required H_2_O was quantitatively delivered by a peristaltic pump, and the generated steam was preheated to 200 °C via a line heater before being blended with other gases prior to entering the high-temperature furnace. For each test, 200 ± 1 g of the prepared coke was loaded into a reaction tube. The reaction tube, constructed of GH3044 high-temperature superalloy steel with a diameter of 74 mm and a length of 800 mm, was suspended beneath an electronic balance using hooks, with gas inlet and outlet ports located on the left and right sides, respectively. The mixed gas was synthesized from various gas cylinders, and the required gas composition for the experiments was obtained by regulating the flow rates of the individual gases.

After gasification, the splitting tensile strength of coke reacted under each atmosphere was measured using the strength testing apparatus illustrated in Figure 1b. The coke particle was positioned horizontally, and a compressive load was applied diametrically; the induced transverse tensile stress fractured the coke, and the maximum fracture load was recorded. Equations (1) and (2) were used to calculate the splitting tensile strength and gasification reaction rate of coke after gasification under different atmospheres, respectively.

(1) $$\sigma =\frac{2P}{\pi dl}$$

where *σ* is the splitting tensile strength (GPa), *P* is the maximum fracture load (N), *d* is the coke-specimen diameter (mm), and *l* is the coke-specimen length (mm).

(2) $$R_{c}=\frac{M_{1}-M_{2}}{M_{1}}\times 100\%$$

where *M*_1_ is the coke mass before gasification (g), *M*_2_ is the residual coke mass after gasification (g), and *R_c_* is the coke gasification reaction rate (%).

Table 3 lists the gas compositions and reaction temperature profiles implemented in the experiments. During the experimental process, the charge was initially heated to 900 °C at a heating rate of 9 °C min^−1^ and isothermally held at this temperature for 30 min. Subsequently, the charge was further heated at a rate of 5 °C min^−1^ to the preset termination temperature of 1200 °C. Prior to reaching the predetermined temperatures, the furnace was purged with N_2_ at a flow rate of 5 L min^−1^; thereafter, the inert gas was switched to the reducing gas with a flow rate of 15 L min^−1^. Upon reaching the maximum heating temperature of 1200 °C, the heating program was shut down, and the atmosphere was switched back to N_2_ to allow the sample to cool to room temperature. Once cooled, the sample was retrieved and weighed.

#### 2.2.3. Soft-Melting Behavior

**Figure 2:**
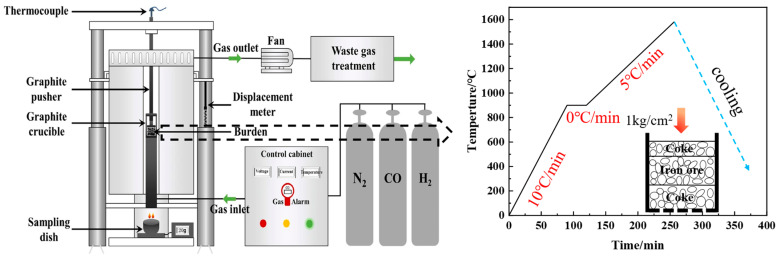
Schematic diagram of softening–melting furnace.

The experimental arrangement and operating sequence are summarized in Figure 2. For each run, a three-layer bed was assembled in a graphite crucible with an internal diameter of 94 mm and a height of 210 mm. An 80 g coke layer was placed at the bottom, followed by 500 g of ferrous burden and a 40 g upper coke layer. The initial thickness of the packed bed was recorded, after which an axial compressive stress of 1 kg/cm^−2^ was applied. Furnace temperature, bed contraction, and pressure drop were monitored continuously throughout the test. The required atmospheres were prepared from individual gases of 99.99% purity in a gas-mixing cabinet. Four temperature-dependent reducing-gas programs were generated using the multizone-constrained mathematical model [31,32], and the corresponding compositions are reported in Table 4. Before the furnace reached 200 °C, N_2_ was introduced at 5 L min^−1^; above 200 °C, it was replaced by the selected reducing-gas mixture at 12 L min^−1^. The burden was heated to 900 °C at 10 °C min^−1^, maintained at this temperature for 30 min to reproduce the thermal reserve zone, and then heated at 5 °C min^−1^. The run was terminated when the cumulative mass of the dripped product reached 20 g. Heating was then stopped, N_2_ was restored at 5.0 L min^−1^, and the specimen was cooled to room temperature under protective gas.

The characteristic temperatures were extracted from the bed-contraction curve, the pressure-drop response, and the observation of liquid discharge. T_10_ and T_40_ correspond to the temperatures at which the packed bed has contracted by 10% and 40%, respectively. The melting-start temperature, Ts, was assigned to the onset of the sustained sharp rise in pressure drop, whereas the dripping-start temperature, Td, was recorded when molten iron first emerged from the crucible. The softening zone (ΔT_S_), melting zone (ΔT_M_), and total cohesive-zone interval (ΔT_SM_) were subsequently obtained from T_40_ − T_10_, T_d_ − Ts, and T_d_ − T_10_, respectively. The highest pressure drop measured during a run is reported as ΔPmax.

Gas-flow obstruction over the melting and dripping stage was evaluated from the area under the pressure-drop–temperature curve between Ts and T_d_. This cumulative quantity is defined as the permeability index S and was calculated using Equation (3):

(3) $$S=\int_{T_{s}}^{T_{d}}pdT$$

where *S* is the permeability index (kPa·°C) and *p* is the pressure difference in the ferrous burden.

## 3. Results and Discussion

### 3.1. Analysis of the Onset Behavior of Coke Solution Loss Reaction

**Figure 3:**
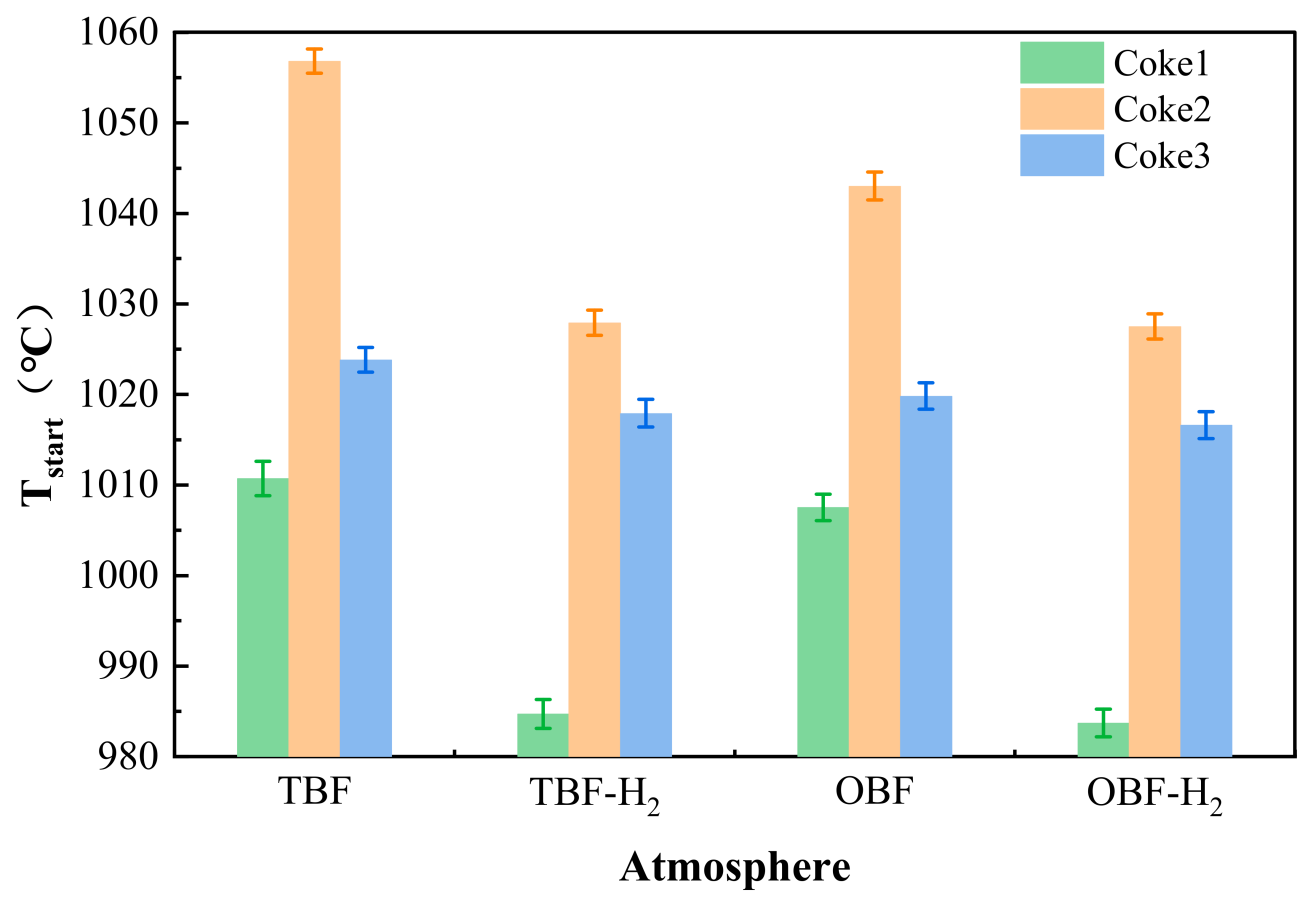
Reaction start temperatures of coke solution loss under different atmospheres.

As illustrated in Figure 3, the reaction initiation temperature of coke solution loss is jointly governed by the intrinsic properties of the coke itself and the prevailing blast furnace atmosphere. The CRI of the coke is the principal intrinsic factor governing this temperature. Under each blast-furnace atmosphere, the reaction initiation temperature of the coke decreases monotonically as the CRI value increases. Under the TBF atmosphere, the reaction initiation temperature for Coke 1 is 1056.8 °C, whereas the initiation temperatures for Coke 2 and Coke 3 decrease to 1023.8 °C and 1010.7 °C, respectively, yielding a maximum temperature differential of 46.1 °C. This behavior arises because the CRI value inherently depends on the microstructural ordering and the active site density of the carbonaceous matrix within the coke. Highly reactive cokes (with higher CRI values) possess an increased proportion of amorphous carbon and optically isotropic structure, characterized by smaller carbon crystallites and abundant lattice defects. These structural features provide a high density of active sites on the coke surface, thereby lowering the chemical activation energy of the gasification reaction. Consequently, the reactant gas molecules can overcome the energy barrier of gasification at a lower temperature. Conversely, low-CRI cokes exhibit a structural configuration that tends toward a highly graphitized matrix with strong lattice binding energy. This structural integrity imparts enhanced thermochemical inertness against gaseous attack and therefore requires a higher-temperature zone to activate the solution loss reaction.

For a given coke, the reaction initiation temperatures under the different atmospheres consistently followed the order TBF > OBF > TBF-H_2_ > OBF-H_2_. Among the atmosphere-related factors, hydrogen enrichment was the principal external factor lowering the reaction initiation temperature. After H_2_ was introduced into the TBF atmosphere, the reaction initiation temperatures of Cokes 1 and 3 decreased by 28.9 and 26.0 °C, respectively. This trend occurs because coke gasification in a hydrogen-enriched blast furnace changes from a predominantly C–CO_2_ reaction to the combined contribution of C–CO_2_ and C–H_2_O reactions. The kinetic diameter of H_2_O is smaller than that of CO_2_, which reduces its internal diffusion resistance within coke micropores. In addition, the apparent activation energy of C–H_2_O gasification is lower than that of the C–CO_2_ reaction. Together, these factors allow surface adsorption, reaction, and desorption to begin at a lower temperature. In the OBF atmosphere, removal of most non-reactive N_2_ also increases the partial pressures of the reactive gases. According to the law of mass action for heterogeneous reactions, the higher reactant concentration increases the effective collision frequency between gas molecules and active sites on the coke skeleton, further shifting the reaction initiation temperature to a lower value.

### 3.2. Coke Reaction Behavior

#### 3.2.1. Gasification Behavior and Compressive Strength of Coke

**Figure 4:**
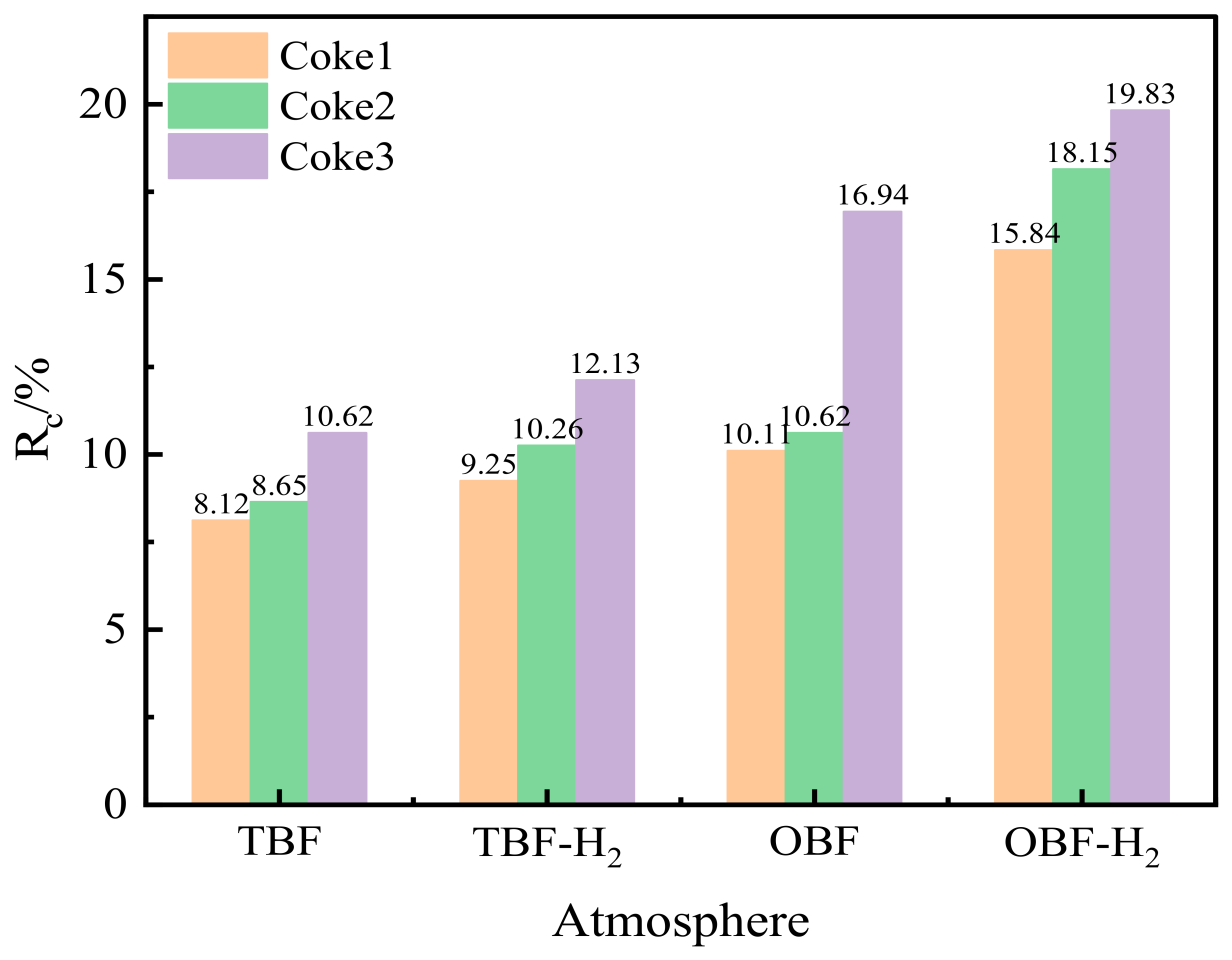
*R_C_* of cokes under different atmospheres.

Figure 4 shows the coke gasification reaction rate (*R_c_*) of cokes with different CRI under various atmospheres. The results show that under the TBF, TBF-H_2_, OBF, and OBF-H_2_ conditions, *R_c_* increases with intrinsic coke reactivity. This is because a higher coke reactivity corresponds to a less ordered carbon structure and a greater abundance of active sites, which facilitates the carbon solution loss reaction and consequently increases the extent of gasification. Furthermore, when hydrogen is injected into the TBF and OBF, the *R_c_* values of the three experimental cokes with different CRI all increase. Among them, Coke 3, which possesses the highest CRI, displays the most prominent increase; compared to the TBF condition, its *R_c_* increases by 1.51 percentage points under TBF-H_2_, and the RC increment reaches 2.89 percentage points under OBF-H_2_. This trend is consistent with previous studies showing that H_2_/H_2_O-containing atmospheres accelerate coke gasification and increase coke mass loss under simulated blast furnace conditions [13,14]. Crucially, the enhancing effect of OBF-H_2_ on coke gasification is more pronounced, with its impact significantly surpassing that of TBF-H_2_. This is closely associated with the higher concentration of active gaseous components and more favorable reaction kinetics in the OBF-H_2_ atmosphere.

**Figure 5:**
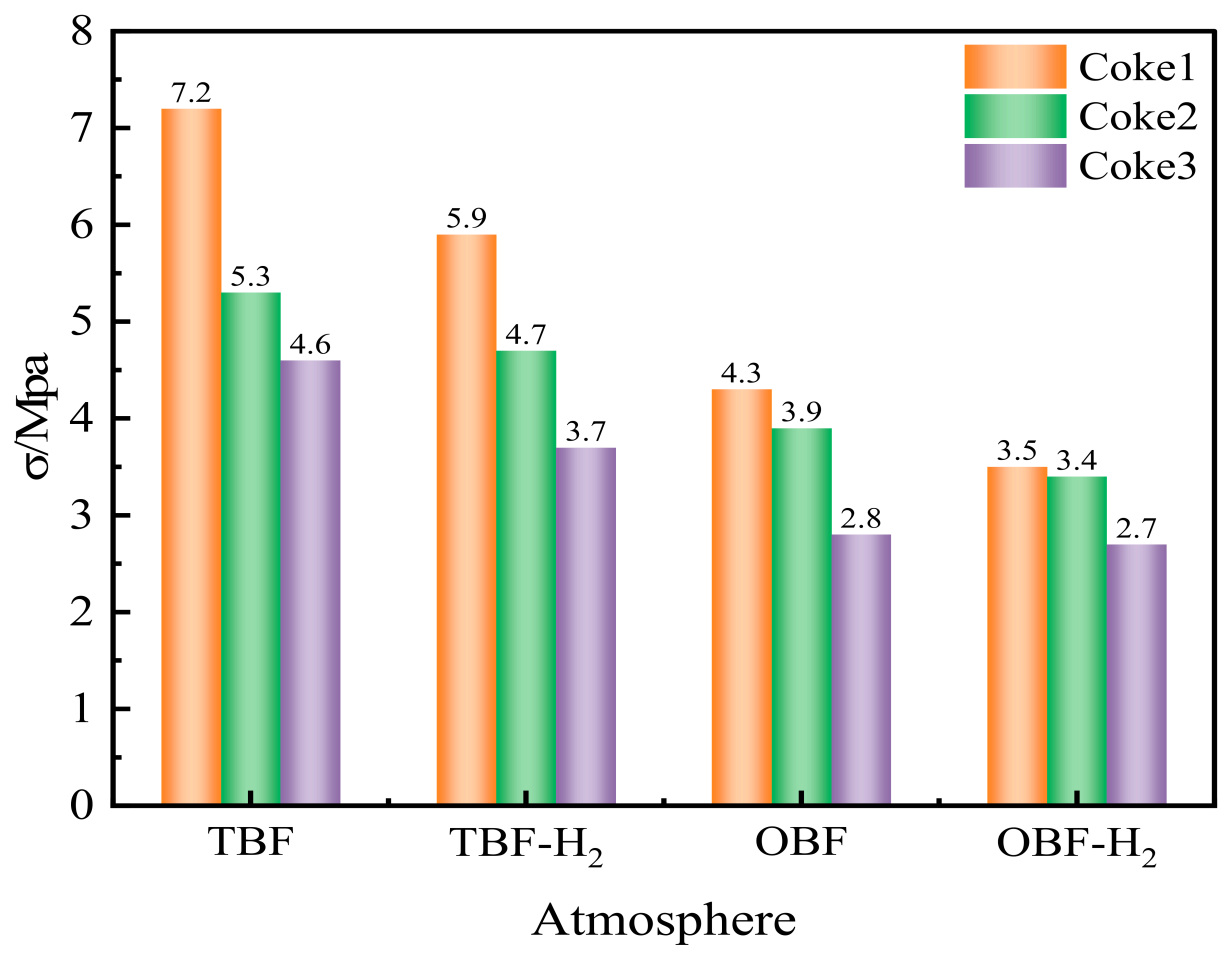
Compressive strength of coke after reaction under different atmospheres.

Differences in coke reaction behavior lead to differences in post-reaction strength. Figure 5 illustrates the splitting tensile strength results of cokes with different CRIs after gasification under various atmospheres. The results show that across all four atmospheres, the splitting tensile strength of the cokes decreases progressively as intrinsic CRI increases. Following hydrogen injection into both the TBF and OBF, the splitting tensile strength of the three experimental cokes exhibits a further declining trend. Specifically, for Coke 3, which possesses the highest reactivity, the splitting tensile strength drops by 0.9 MPa and 0.1 MPa after reacting under the TBF-H_2_ and OBF-H_2_ conditions, respectively. These results indicate that the detrimental impact of OBF-H_2_ on the mechanical strength of coke is lower than that of TBF-H_2_. Nonetheless, the TBF-H_2_, OBF, and OBF-H_2_ atmospheres promote coke gasification and pore development, which can reduce the effective load-bearing cross-section and post-reaction strength of the coke [13,33].

#### 3.2.2. Evolution Characteristics of Coke Micro-Morphology

**Figure 6:**
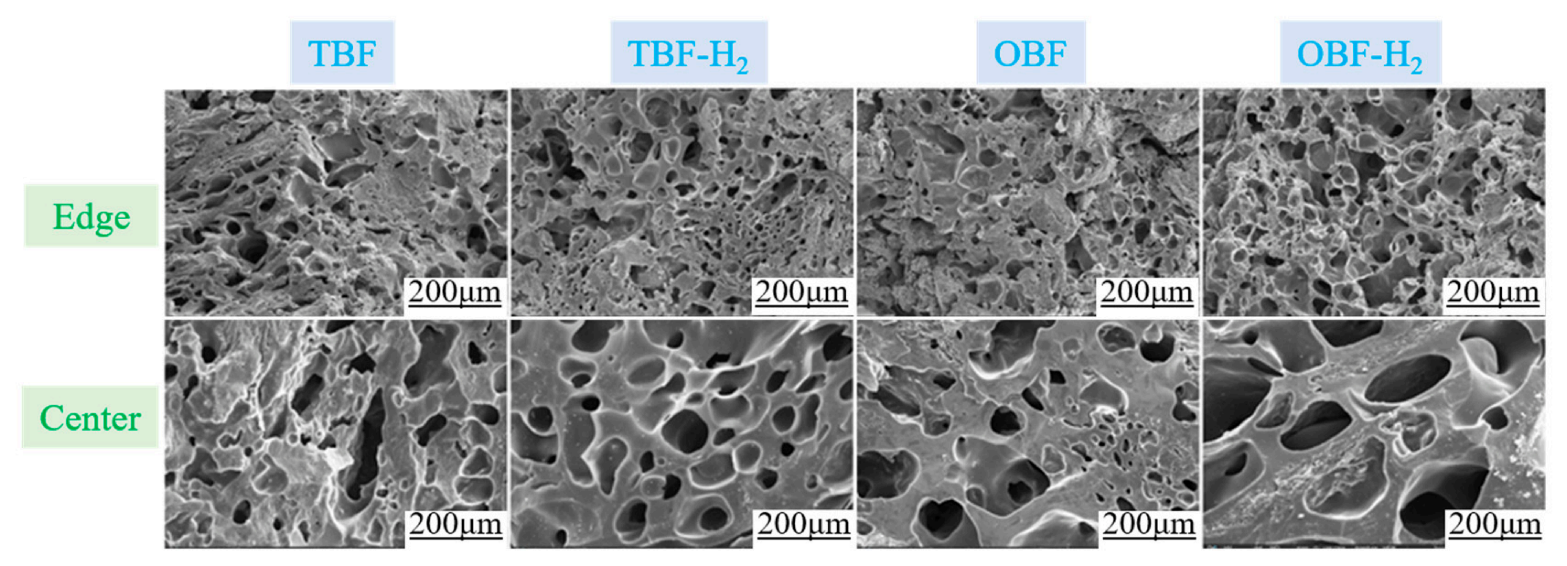
Microscopic morphology of Coke 2 under different atmospheres.

To investigate the effects of different gas compositions on the coke gasification reaction, the micromorphologies of Coke 2 samples after reacting under TBF, TBF-H_2_, OBF, and OBF-H_2_ atmospheres were observed, as shown in Figure 6. A clear difference is observed in the pore distribution between the periphery and the core of the coke, with the pores generated at the periphery being notably larger than those at the core. Under the TBF atmosphere, the core of the post-reaction coke exhibits minimal erosion, whereas the peripheral region is severely eroded, causing small pores to expand into macropores and forming numerous interconnected pore networks. As the reduction potential increases, the H_2_O-containing atmosphere may promote gasification within the connected fine-pore network of the coke. Previous studies have reported that H_2_O/CO_2_ mixtures generate additional small pores within coke, while reaction-diffusion analysis has indicated that H_2_O can exhibit a higher effective internal diffusion coefficient than CO_2_ and promote the connection of pore walls in deeper pore regions [13,33,34]. In agreement with these findings, the increased number of pores in the coke core, the thinning and local coalescence of pore walls, and the more uniform radial pore distribution observed in Figure 6 suggest a more spatially distributed weakening pattern under hydrogen-rich conditions. However, because the penetration depth and local reaction rate were not measured directly, the present micrographs cannot independently demonstrate deeper molecular penetration of H_2_O.

**Figure 7:**
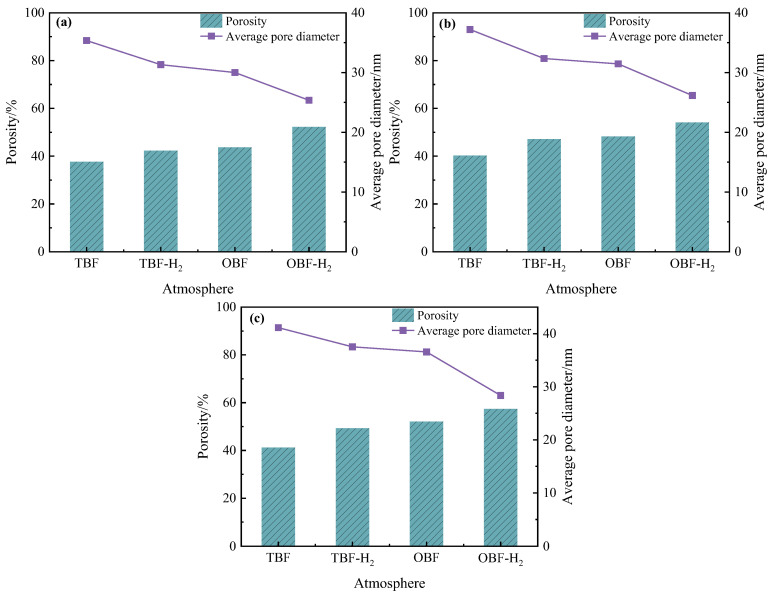
Porosity and average pore diameter of cokes after reacting under different atmospheres: (**a**) Coke 1; (**b**) Coke 2; (**c**) Coke 3.

The pore-structure characteristics of the cokes after reacting under different atmospheric conditions are illustrated in Figure 7. Across the TBF, TBF-H_2_, OBF, and OBF-H_2_ atmospheres, the porosities of all three cokes increase progressively. Specifically, the porosity increases by 13.32% for Coke 1 and 13.90% for Coke 2, whereas Coke 3, which possesses the highest reactivity, shows the largest increase of 16.13%. Thus, the increase in porosity becomes larger as coke reactivity increases. However, in the presence of H_2_O, the average pore diameter of the three cokes decreases. Previous studies have shown that H_2_O-containing atmospheres promote the formation and development of fine internal pores, whereas CO_2_-dominated gasification more strongly enlarges pre-existing pores; the resulting pore evolution depends on temperature, conversion, and the initial pore network [13,33,34]. Consequently, although the gasification reaction rates under the TBF-H_2_, OBF, and OBF-H_2_ atmospheres are higher than those under the TBF atmosphere, their corresponding average pore diameters decrease. Compared with the TBF and OBF conditions, the hydrogen-rich conditions lead to a higher porosity in the cokes, which further suppresses their average pore diameter.

#### 3.2.3. Evolution of Coke Optical Structure

The typical optical structure of coke comprises isotropic (I), fine mosaic (*Mf*), medium mosaic (*Mm*), coarse mosaic (*Mc*), incompletely fibrous (*Fi*), fully fibrous (*F*), leaf-like (*L*), and inert/fused fragments (FF). Table 5 lists the optical property parameters of the raw cokes and the post-reaction cokes under TBF, TBF-H_2_, OBF, and OBF-H_2_ atmospheres. Here, (I + FF) represents the highly isotropic microstructure, whereas (Mf + Mm + Mc) denotes the highly anisotropic microstructure. The optical texture index (OTI) reflects the degree of coke anisotropy, which was calculated according to Equation (4).

(4) OTI=1∗Mf+1.5∗Mm+2∗Mc+2.5∗Fi+3∗F+4∗L

Table 5 demonstrates that the experimental cokes exhibit high proportions of I, Mf, Mm, fibrous structure, and FF. From the original coke to the coke reacted under the OBF-H_2_ atmosphere, the proportion of FF increases from 34.9% to 56.8%, corresponding to an increase of 21.9%, whereas the proportion of Mm decreases from 34.9% to 21.1%, corresponding to a decrease of 13.8%. Previous studies have demonstrated that the gasification rates of different optical structures are not identical. Under CO_2_ conditions, isotropic and fine mosaic structures generally exhibit higher reactivity than coarse mosaic, fibrous, and leaf-like structures [35,36]. In addition, in situ observations have shown that coarse mosaic and flow structures exhibit lower resistance to H_2_O than to CO_2_, while H_2_O gasification can cause more pronounced pore development and structural deterioration [33,36]. Thus, the decrease in Mm and the increase in FF observed in the present study are consistent with the preferential consumption or fragmentation of mosaic domains under H_2_O-containing conditions. Nevertheless, optical microscopy alone cannot distinguish selective chemical consumption from the mechanical fragmentation of previously continuous anisotropic domains.

**Figure 8:**
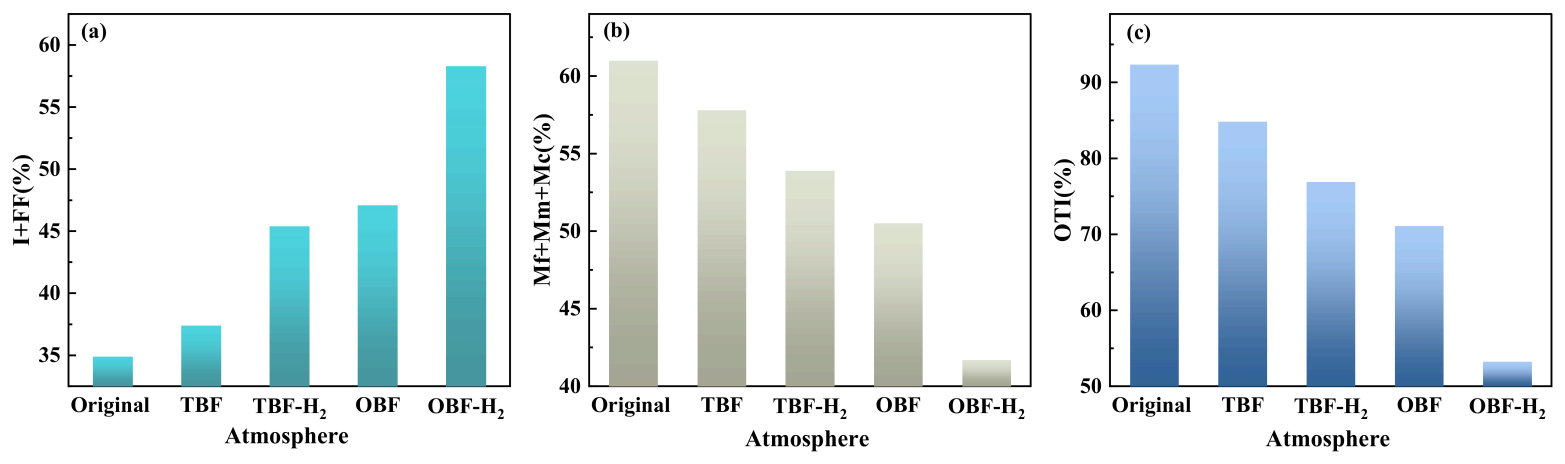
Optical structure evolution of the original and post-reaction coke: (**a**) isotropic fraction, I + FF; (**b**) mosaic anisotropic fraction, Mf + Mm + Mc; (**c**) optical texture index (OTI).

Figure 8 shows the changes in (I + FF), (Mf + Mm + Mc) and the OTI of the raw and post-reaction cokes under different atmospheres. As shown in Figure 8a,b, as the reduction potential increases, the content of (I + FF) within the coke matrix increases continuously, whereas that of (Mf + Mm + Mc) exhibits a steady decline. As the reduction atmosphere transitions from TBF to OBF-H_2_, the concentration of steam (H_2_O), in particular, increases significantly. Previous studies have shown that H_2_O gasification promotes pore development, reduces post-reaction coke strength, and can produce more pronounced structural deterioration than CO_2_ gasification [13,33]. Reaction-diffusion analysis further indicates that H_2_O can exhibit a higher effective internal diffusion coefficient than CO_2_ under comparable conditions, while gasification-induced changes in pore structure and coke-matrix mechanical properties can influence crack initiation and skeleton strength [34,37]. Accordingly, the decrease in the strength-bearing anisotropic fraction (Mf + Mm + Mc) from 61.0% to 41.7%, together with the increase in FF from 34.9% to 56.8%, is consistent with enhanced reaction and fragmentation of the anisotropic load-bearing domains under the OBF-H_2_ atmosphere. The corresponding decrease in OTI from 92.4 to 53.3 further indicates substantial degradation of the coke optical texture. However, because the present study did not directly measure H_2_O penetration depth, spatially resolved reaction rates, microcrystalline bond cleavage, or fracture propagation, these results should not be interpreted as direct proof of a complete transition from localized surface gasification to uniform full-dimensional skeleton degradation.

### 3.3. Softening–Melting Behavior of the Ferrous Burden

**Figure 9:**
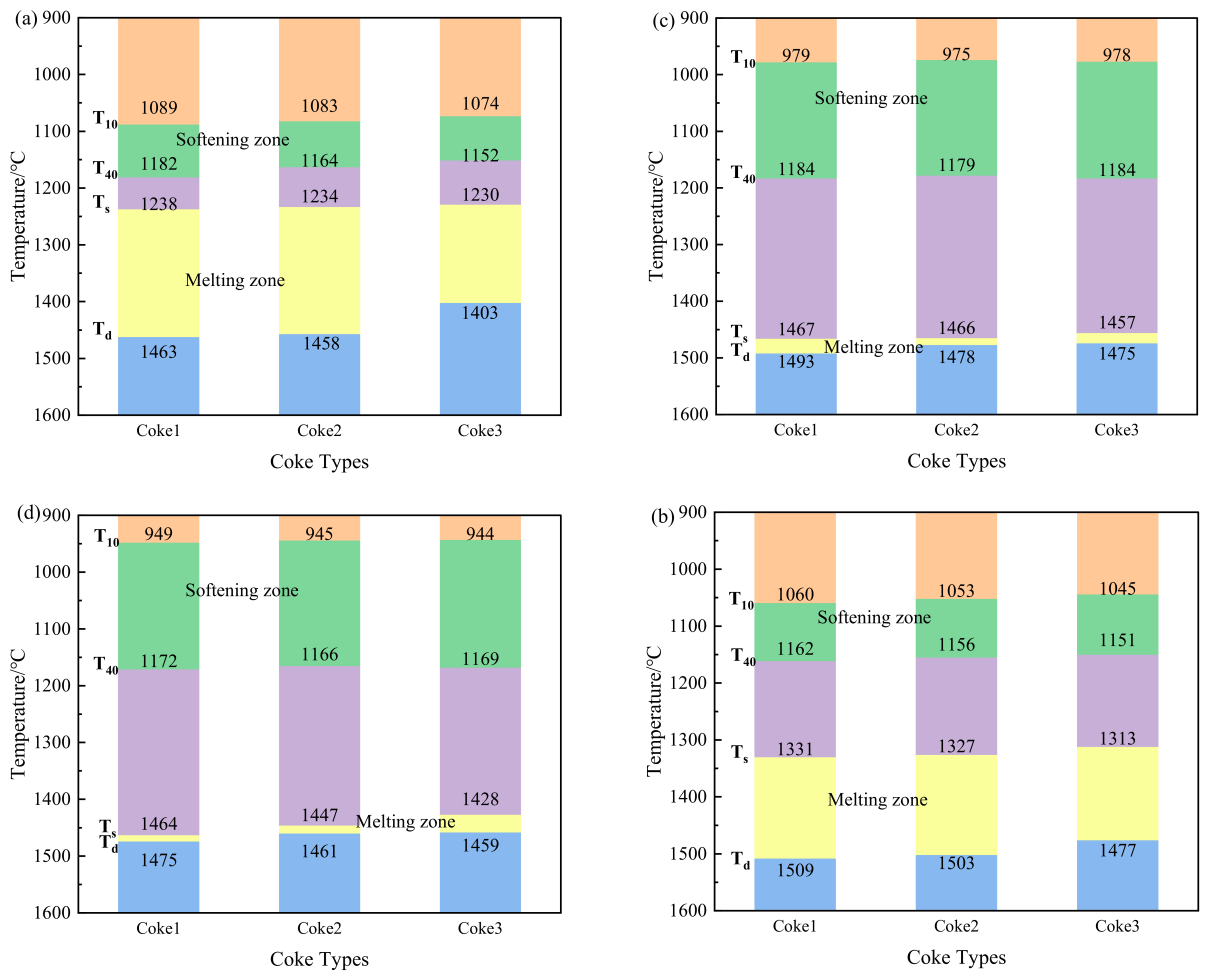
Effects of coke reactivity under different atmospheres on the softening and melting behavior of the burden: (**a**) TBF; (**b**) TBF-H_2_; (**c**) OBF; (**d**) OBF-H_2_.

The influence of coke reactivity under different atmospheres on the softening and melting behavior of the ferrous burden is illustrated in Figure 9. The results show that under the TBF and TBF-H_2_ atmospheres, both the T_10_ and T_40_ of the ferrous burden decrease progressively as coke reactivity increases. Specifically, under the TBF atmosphere, T_10_ decreases from 1089 °C to 1074 °C, whereas under the TBF-H_2_ atmosphere, T_10_ drops from 1060 °C to 1045 °C. This trend occurs because highly reactive cokes intensify the solution loss reaction, thereby generating high concentrations of CO and H_2_. These highly reducing gases promote the early-stage reduction of the iron-bearing burden, leading to the formation of a large amount of wüstite. The accumulation of FeO subsequently lowers the melting point of the primary slag phase, thereby lowering T_10_. However, under the OBF and OBF-H_2_ atmospheres, because most N_2_ dilution is eliminated, the gas reduction potential is significantly enhanced, which accelerates the reduction rate of the burden. Consequently, the influence of coke reactivity on T_10_ is weaker under these full-oxygen conditions.

Coke reactivity exerts a significant regulatory effect on the melting and dripping behavior of the burden. With the increase in coke reactivity, both Ts and T_d_ under all four atmospheres exhibit a progressive decline. For instance, under the TBF atmosphere, T_d_ decreases from 1463 °C to 1403 °C as reactivity increases. This trend occurs because higher coke reactivity increases the degree of gasification at the coke periphery, resulting in a high local accumulation of ash. This ash layer acts as a physical barrier that hinders the direct reduction of FeO within the molten slag by the underlying coke matrix, thereby lowering Ts. Consequently, a relatively high residual FeO content is retained in the molten slag. This improves the slag’s melting and flow properties, allowing it to combine with the ash on the surface of the coke. This continuous surface renewal subsequently promotes the occurrence of the coke-iron carburization reaction, thereby contributing to the reduction of T_d_.

**Figure 10:**
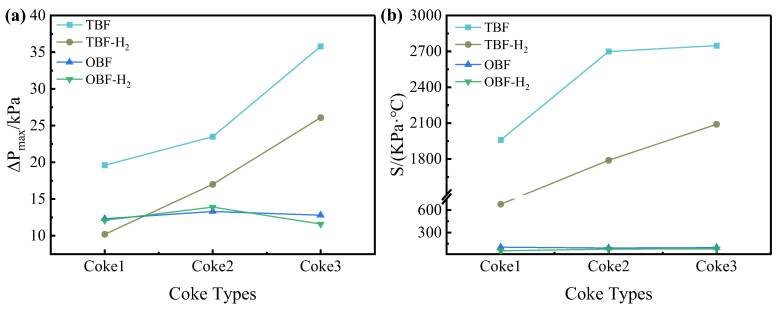
Effects of coke reactivity under different atmospheres on the permeability of the ferrous burden: (**a**) maximum pressure drop, ΔPmax; (**b**) permeability index, S.

The permeability of the ferrous burden under different conditions is illustrated in Figure 10. The results show that under the TBF atmosphere, as coke CRI increases, the ΔPmax increases from 19.5 kPa to 35.7 kPa, and the S value increases from 1950 kPa·°C to 2750 kPa·°C. Under the TBF-H_2_ atmosphere, although the overall permeability of the burden column is enhanced owing to the high reduction potential of H_2_, the ΔPmax still rises from 10.2 kPa to 26.1 kPa, and the S value increases from 650 kPa·°C to 2090 kPa·°C as coke reactivity increases. This result indicates that, in traditional process systems, the severe solution-loss reaction of highly reactive cokes in the high-temperature cohesive zone causes critical degradation and pulverization of the coke skeleton. It also triggers premature and excessive exudation of low-melting primary slag, thereby severely impairing burden permeability. In contrast to the TBF condition, the variations in coke reactivity exert a negligible impact on the burden permeability under both the OBF and OBF-H_2_ atmospheres. Under these full-oxygen conditions, as the coke reactivity increases from Coke 1 to Coke 3, the ΔPmax is maintained within a narrow range of 11.5–14.0 kPa. The S value also remains below 150 kPa·°C under all coke conditions.

### 3.4. Coke Adaptability Under Different Process Atmospheres

By combining the reaction behavior, microstructural evolution, and cohesive-zone permeability resistance of coke under different atmospheres, the engineering adaptability of cokes with different reactivities can be evaluated for each blast-furnace process. Under the TBF atmosphere, burden-column permeability is highly sensitive to coke reactivity. For the highly reactive Coke 3, the reaction initiation temperature decreases to 1010.7 °C, shifting measurable gasification to a lower-temperature region. The intensified solution-loss reaction in the lower high-temperature zone then causes severe gasification-induced degradation and a marked increase in pressure drop, indicating poor process adaptability. Consequently, the TBF atmosphere requires a high-quality coke such as Coke 1, which has a low CRI and high CSR, to maintain the mechanical support provided by the coke skeleton. Under TBF-H_2_, the high reduction potential of H_2_ partly alleviates flow resistance caused by slag–iron holdup. However, the high H_2_O concentration promotes reaction within connected pores, degrades anisotropic structure, and accelerates the consumption of Mm. The resulting weakening is therefore distributed more broadly through the coke skeleton. Highly reactive coke remains unsuitable for this condition, whereas Coke 2 exhibits better adaptive stability because of its temperature-passivation characteristics during the atmospheric transition. The process response changes markedly under OBF and OBF-H_2_. Although the H_2_O-containing atmosphere causes severe microscopic texture fragmentation, as indicated by the increase in I + FF to 58.3% and the decrease in OTI, removal of most N_2_ produces a high gas-reduction potential. The ferrous burden can therefore reach a high degree of reduction before entering the melting zone, which narrows the cohesive zone. Consequently, ΔPmax and S remain at low levels for all three cokes. These results indicate that the OBF and OBF-H_2_ atmospheres alter the fluid-dynamic behavior of the cohesive zone and, under the present laboratory conditions, reduce the conventional dependence on high coke thermal strength.

It should be noted that while these laboratory-scale results provide mechanistic insight into the process tolerance of OBF/OBF-H_2_ to reactive cokes, direct industrial implementation requires further investigation. Comprehensive evaluations involving process simulation, pilot-scale trials, and economic analyses are necessary to confirm long-term operational stability and cost-effectiveness under actual blast furnace conditions.

## 4. Conclusions

(1) The reaction initiation temperature of the coke solution-loss reaction is jointly governed by intrinsic coke properties and the prevailing gas atmosphere. Under the same atmosphere, the reaction initiation temperature decreases progressively as CRI increases, with a maximum difference of 46.1 °C among the three cokes. A hydrogen-enriched atmosphere introduces C–H_2_O gasification in addition to the C–CO_2_ reaction and lowers the initiation temperature by 26.0–28.9 °C. This shift to a lower temperature is most pronounced under OBF-H_2_.
(2) The high H_2_O concentration in hydrogen-rich atmospheres substantially alters the gasification-induced degradation mechanism of the coke matrix. H_2_O-containing atmospheres promote reaction within connected pores and intensify the degradation and fragmentation of anisotropic coke structure. Consequently, the proportion of the strength-bearing anisotropic structure drops significantly from 61.0% to 41.7%, and the OTI value decreases from 92.4 to 53.3. These results indicate more spatially distributed structural degradation; however, they do not by themselves prove uniform H_2_O penetration or complete skeleton disintegration.
(3) The effects of coke reactivity on the softening–melting behavior and permeability of the ferrous burden depend strongly on the specific blast furnace technological system. Under the TBF process, the burden permeability is highly sensitive to coke reactivity; highly reactive cokes intensify the solution loss within the high-temperature zone and promote the exudation of low-melting-point slag phases, increasing ΔPmax to 35.7 kPa. Conversely, under the OBF and OBF-H_2_ atmospheres, the cohesive zone narrows and shifts downward, allowing both ΔPmax and the S value to be maintained at low levels across all coke conditions.
(4) Oxygen blast furnace technologies provide an additional basis for evaluating the engineering adaptability of metallurgical coke. Under the present laboratory conditions, OBF atmospheres reduced the sensitivity of cohesive-zone permeability to coke thermal strength, supporting further evaluation of highly reactive cokes in low-carbon ironmaking processes.

## Figures and Tables

**Table 1:** Chemical composition of the ferrous burden (wt.%).

Chemical Compositions	TFe	FeO	SiO_2_	Al_2_O_3_	CaO	MgO
Acid pellet	61.94	0.19	7.50	2.59	0.50	0.48

**Table 2:** Industrial analysis, CRI, and CSR of the coke samples.

Sample	Industrial Analysis (wt.%)			CSR (%)	CRI (%)
	F_C_	Volatile	Ash		
Coke 1	78.15	3.33	18.11	66.6	20.7
Coke 2	81.33	2.81	15.44	62.4	26.4
Coke 3	80.5	7.85	14.73	45.2	40.3

Note: F_C_, fixed carbon; Volatile, volatile matter; Ash, ash content; CSR, coke strength after reaction; CRI, coke reactivity index.

**Table 3:** Coke gasification reaction temperature and gas composition.

Sample	Temperature (°C)	Atmosphere	Gas Composition (vol.%)				
			N_2_	CO	CO_2_	H_2_	H_2_O
Coke 1 Coke 2 Coke 3	800–1000 °C	TBF	53.81	20.92	21.08	2.61	1.58
		TBF-H_2_	53.44	14.84	16.77	10.36	4.58
		OBF	8.23	47.34	30.25	7.28	6.90
		OBF-H_2_	2.24	35.53	28.98	21.62	11.63
	1000–1050 °C	TBF	59.49	25.13	10.75	2.89	1.74
		TBF-H_2_	49.50	26.54	10.12	8.62	5.22
		OBF	8.17	54.71	23.04	7.03	7.04
		OBF-H_2_	2.19	46.49	18.80	16.29	16.21
	1050–1100 °C	TBF	59.68	26.33	9.34	3.03	1.62
		TBF-H_2_	49.71	27.71	8.68	9.26	4.63
		OBF	8.05	58.15	19.92	7.28	6.60
		OBF-H_2_	2.14	51.73	14.38	16.88	14.85
	1100–1200 °C	TBF	54.39	33.81	7.55	3.08	1.16
		TBF-H_2_	54.39	26.54	3.85	11.43	3.77
		OBF	8.07	58.07	19.93	7.12	6.79
		OBF-H_2_	1.96	61.15	7.87	21.30	7.72

**Table 4:** Experimental parameters of softening–melting behavior [31,32].

Condition	Parameters							
Temperature (°C)	200–750				750–900			
Gas composition (vol.%)	H_2_	CO	CO_2_	N_2_	H_2_	CO	CO_2_	N_2_
TBF	3.2	21.3	20.0	55.5	3.7	25.4	13.3	57.6
TBF-H_2_	10.0	17.0	18.0	55.0	12.4	21.1	12.0	54.5
OBF	10.0	45.9	36.2	7.9	11.6	58.4	24.1	5.9
OBF-H_2_	23.3	31.4	31.1	14.2	27.4	42.2	20.7	9.7
Temperature (°C)	900				900–1580			
Gas composition (vol.%)	H_2_	CO	CO_2_	N_2_	H_2_	CO	CO_2_	N_2_
TBF	4.2	29.4	6.6	59.8	4.7	33.5	0.0	61.8
TBF-H_2_	14.8	25.2	6.0	54.0	17.1	29.2	0.0	53.7
OBF	13.2	70.9	12.0	3.9	14.7	83.4	0.0	1.9
OBF-H_2_	31.5	53.0	10.3	5.2	35.7	63.7	0.0	0.6

**Table 5:** Optical structural fractions and OTI values for original coke and post-reaction coke.

Sample	Optical Structure										OTI
	I	Mf	Mm	Mc	Fi	F	L	FF	I + FF	Mf + Mm + Mc	
Original coke	1.2	20.9	34.9	5.2	2.9	0	0	34.9	36.1	61.0	92.4
TBF	1.8	24.7	30.1	3	3	0	0	37.4	39.2	57.8	84.9
TBF-H_2_	3.6	24.4	27.4	2.1	2.4	0	0	43.5	47.1	53.9	76.9
OBF	4.2	18.2	27.6	4.7	0.7	0	0	41.2	45.4	50.5	71.1
OBF-H_2_	1.5	19.6	21.1	1.0	0.0	0	0	56.8	58.3	41.7	53.3

## Data Availability

The original contributions presented in this study are included in the article. Further inquiries can be directed to the corresponding author.
